# Dual facets of MSC-derived small EVs: regulatory insights into antitumor mechanisms in pancreatic ductal adenocarcinoma

**DOI:** 10.1007/s12032-025-02713-5

**Published:** 2025-04-10

**Authors:** Demet Kaçaroğlu, Seher Yaylacı, Alper Murat Ulaşlı

**Affiliations:** 1 Faculty of Medicine, Department of Medical Biology, Lokman Hekim University, Söğütözü, 2179. Sk. No:6, 06530 Çankaya, Ankara Turkey; 2 Stem Cell Institute, Interdisciplinary Stem Cell and Regenerative Medicine Department, Ankara University, Cevizlidere, Ceyhun Atuf Kansu Cd. No:169, 06520 Çankaya, Ankara Turkey

**Keywords:** Mesenchymal stem cells, PDAC, Small EVs, Pancreatic cancer, Cancer therapy

## Abstract

Pancreatic ductal adenocarcinoma (PDAC) is characterized by a dense, fibrotic, immunosuppressive, and desmoplastic extracellular matrix. Mesenchymal stem cell (MSC)-derived extracellular vesicles (EVs) have emerged as a novel therapeutic strategy. Nonetheless, the potential dual effects of MSC-EVs on tumor cells warrant careful consideration. This study aimed to evaluate the mechanistic effects of MSC-EVs on PDAC. Wharton’s Jelly (WJ) MSC-derived small EVs were isolated using ultracentrifugation method and analyzed through nanoparticle tracking analysis (NTA) and flow cytometry. EVs were added to Panc-1 cells at concentrations of 4000–10,000 EVs per cell, and a preliminary MTT assay was performed. In subsequent experiments, EVs were added to Panc-1 cells at concentrations of only 4000, 8000 and 12,000 EVs per cell. After 24 h, apoptosis and cell cycle analyses were performed. The expression of epithelial-mesenchymal transition (EMT)-related and immune-related genes was analyzed. Cell cycle analysis showed higher G1 phase percentage in the control group (31%) compared to MSC EV-treated groups (35–36%). Apoptosis analysis revealed similar viable and necrotic cell percentages among the control (80% viable) and treated groups (approximately 78–79% viable). The CD44, VIM, MMP9, TIMP1, and ZEB1 genes were downregulated in treated groups compared to the control. Although CLDN1 and CDH1 genes were upregulated at the lowest EV concentration, they were downregulated at higher EV concentrations. Immune gene analysis showed downregulation of pro-inflammatory cytokines (IL-6, TNF-α, IFN-γ, IL-1α, IL-1β) and upregulation of the anti-inflammatory cytokine IL-10 in treated groups. This study revealed the dual role of WJ-MSC small EVs in PDAC. While they suppressed cell proliferation and modulated EMT markers, indicating their antitumor potential, they also exhibited an immunosuppressive profile. These findings highlight both the promise and challenges of using WJ-MSC small EVs as therapeutic agents, necessitating further studies to optimize their application and balance their effects.

**Supplementary Information:**

The online version contains supplementary material available at 10.1007/s12032-025-02713-5.

## Introduction

Pancreatic cancer is the seventh leading cause of cancer-related deaths worldwide and is projected to become the second deadliest cancer by 2030 due to its high mortality rate [1]. Pancreatic cancer is characterized with aggressive progression and a high rate of metastasis; thus, survival rate is low with classical treatments [2]. Pancreatic cancer treatment consists mainly of four methods: surgical treatments, systemic treatments, radiation therapies, and combined treatments. Although surgical resection offers a curative treatment in the early stages, the cancer recurs in approximately 80% of patients who undergo surgery. Since pancreatic ductal adenocarcinoma (PDAC) is highly resistant to current conventional treatment modalities, new treatment strategies are continuously investigated [3]. Treatment failure is frequently attributed to the inability of therapeutic agents to penetrate the dense desmoplastic microenvironment characteristic of pancreatic cancer [4]. Currently, the search for therapies that can both reach cells and decrease resistance to therapy and regulate the microenvironment is ongoing. In this perspective, cell-based therapies are promising, but the adverse immune reaction makes their use difficult. Recently, researchers have suggested that extracellular vesicles (EVs) secreted by stem cells may play a therapeutic role [5].

Mesenchymal stem cells (MSCs) are multipotent stromal cells with high regenerative potential and therapeutic properties, including immunomodulatory, antiapoptotic, pro-angiogenic, and chemoattractant effects [6]. While MSCs demonstrate antitumor effects in both in vitro and in vivo studies, some evidence suggests that they may also support tumor cells [7]. The direct effects of MSCs in cancer therapy vary depending on their source, dose, and the type of cancer being treated. As a result, MSCs are subjected to various modifications and studied as potential cancer therapies [8]. Recently, researchers have investigated the effects of EVs derived from MSCs on tumor progression and their therapeutic potential in cancer therapy [9]. It has been shown that interactions between cancer cells and MSCs regulate critical behaviors in cancer cells, such as proliferation, metastasis, and epithelial–mesenchymal transition (EMT). In addition, MSCs can secrete EVs that modulate the tumor microenvironment [10].

Exosomes, a subtype of EVs, typically sized between 40 and 100 nm, are secreted by all cell types [11]. These vesicles deliver cargo capable of regulating specific genes and proteins in target cells, thereby altering their properties [12]. With these characteristics, exosomes are used for drug transport, the delivery of tumor suppressor agents, and immunomodulatory and anti-inflammatory applications. Moreover, exosomes possess notable properties for cancer therapy, such as targeting capabilities, low immunogenicity, flexibility for modification, and high permeability across the blood–brain barrier [13]. It has been demonstrated that MSC exosomes influence processes such as tumor cell growth, metastasis, apoptosis, angiogenesis, chemosensitivity, and drug resistance [14]. A meta-analysis showed that only 26 and 46% of the studies reported an tumor-suppressive effect for bone marrow MSC-EVs and adipose tissue MSC-EVs, respectively, whereas 88% confirmed a tumor-suppressive role for Wharton’s jelly (WJ-MSC-EVs) [15]. MSCs and MSC-derived membrane microvesicles have demonstrated a strong antitumor effect on pancreatic cancer after treatment with Paclitaxel, due to their capacity to take up and subsequently release the drug [16]. Kamerkar et al. demonstrated that exosomes carrying oncogenic KRAS siRNAs significantly improved survival in mouse models of pancreatic tumors [17]. Exosomes containing miR-145-5p, derived from human umbilical cord MSCs, induced apoptosis by suppressing proliferation in an in vivo pancreatic ductal adenocarcinoma model [18]. In addition, IDO-1-containing exosomes secreted by MSCs limited the immune response by decreasing IFN-γ expression in dendritic cells and NK cells [19]. Although they exhibit tumor-inhibitory properties, tumor-promoting effects of exosomes derived from various MSCs have also been reported in both in vitro and in vivo models of different cancer types [20].

Recent evidence indicates that WJ-MSC-derived EVs may exert both antitumor and pro-tumor effects when applied directly to pancreatic cancer cells, in addition to their well-documented immunomodulatory properties. Notably, WJ-MSC-EVs have shown strong tumor-suppressive capabilities when utilized as carriers for therapeutic agents. Based on these findings, our study aimed to investigate the intrinsic effects of native (unmodified) WJ-MSC-derived EVs on PDAC cells in a dose-dependent manner. Specifically, we focused on evaluating their influence on cell cycle progression, apoptosis induction, epithelial–mesenchymal transition (EMT) modulation, and inflammatory gene expression profiles.

## Material and methods

### Cell cultures and characterization

Pancreatic adenocarcinoma cells (Panc-1/ATCC: CRL-1469) were used after commercial purchase. Cells were cultured in T-25 flasks containing 1% L-Glutamine, 10% Fetal Bovine Serum, 1% penicillin/streptomycin, and high-glucose DMEM (Capricorn, Germany) at 37 °C and 5% CO_2_. MSCs derived from human Wharton’s Jelly (Stembio, Turkey) were purchased commercially and cultured in NutriStem® XF complete medium (Sartorius, Germany) containing 1% L-Glutamine and 1% penicillin/streptomycin at 37 °C and 5% CO_2_. WJ-MSCs were characterized for further experiments. For the detection of CD44, CD90, CD73, and CD105 surface markers on WJ-MSCs, 50,000 cells in a T-75 flask at passage number three were stained using the MSC Marker Verification Kit (R&D Systems, Catalog #FMC020). A positive MSC surface antibody cocktail consisting of 10 μL each of anti-CD90, anti-CD44, anti-CD73, and anti-CD105 was applied. Similarly, a negative control cocktail containing 10 μL each of anti-CD45, anti-CD34, anti-CD11b, anti-HLA-DR, anti-CD79, and 10 μL of isotype control antibody was used.

The mixture was incubated at room temperature in the dark for 30–45 min. Cells were washed with staining buffer and centrifuged to remove any remaining antibodies. Then, the precipitated cell pellet was resuspended in staining buffer for flow cytometry analysis. Cell debris was removed based on forward scatter and side scatter dot plots to select intact cells. Fluorescence results were displayed as histograms using Cellquest Pro software (BD Bioscience, San Jose, CA). The percentages of CD44, CD90, CD73, and CD105 expressed by the cells were determined.

### MSC-Derived small EVs ısolation and characterization

WJ-MSC medium was collected after the culture flasks reached 80–90% confluency to produce concentrated and sufficient amounts of EVs. In the first step of small EVs isolation, WJ-MSC culture medium was centrifuged at 300×*g* for 10 min at 4 °C to remove dead cells. In the second step, the medium was centrifuged at 2500×*g* for 15 min at 4 °C, and the supernatant was transferred to new tubes. In the third step, the medium was centrifuged at 20,000×*g* for 20 min at 4 °C. The supernatant was passed through a 0.22 μm pore size filter to remove microvesicles larger than 220 nm. The pre-purified cell medium was centrifuged at 120,000×*g* for 120 min at 4 °C using a CS150FNX (Hitachi Himac, Japan) ultracentrifuge [21]. After removing the supernatant, the remaining pellets were diluted with 100 μl of PBS. For the characterization of small EVs derived from WJ-MSCs, small EVs surface markers were identified via flow cytometry, and their size and concentration were determined through Nanoparticle Tracking Analysis (NTA). For immunophenotypic cytometry-based EV characterization, EV surface markers were labeled with specific antibodies. The ExoStep™ Kit (Immunostep, Salamanca, Spain) was used for this purpose. Labeled EVs were analyzed on a flow cytometer (Beckman Coulter, California, USA) for marker positivity. CD63 capture antibodies coated on 6 µm diameter magnetic beads served as specific antibodies recognized by the flow cytometer, while CD9 detector antibodies labeled with red fluorescence were used as secondary antibodies, detectable by the flow cytometer’s fluorescence detector. Size and concentration analyses of the small EVs were performed using a NanoSight NTA 3.4 device (Malvern Panalytical, UK). The EVs were stored at − 20 °C for further experiments [21].

### Small EVs protein and cytokine measurement

MSC medium from passage number three was utilized to assess protein and cytokine levels. Isolation was conducted using EXO-Prep HBM-EXP-C25 (HansaBioMed, Estonia) with 1 ml of the medium according to the manufacturer’s instructions. The last pellet was dissolved in 100 µl of PBS. Next, 100 µl of RIPA (Serva, Germany) was added, and protein isolation was performed. A total of 600 µl of protein was prepared for subsequent studies by adding 500 µl of PBS. The ABP Biosciences BCA Protein Assay Kit (Rockville, Maryland) was used for protein quantification with a 20 µl EVs sample. ELISA studies were conducted using ELK Biotechnology kits (Denver, USA) for IL-6, TNF-α, IL-1RA, IL-10, and IL-1β, with 100 µl of sample per well.

### Cell viability analyses

MTT analyses was performed to assess the viability of WJ-MSC-derived exosomes on pancreatic cancer cells. For each experiment, 5000 cells were seeded in 96-well plates for 24 and 48 h evaluation. Six controls were used for each group. After cells adhered, EVs in saline were added to 4000–10,000 exosomes per cell. Each 1 µL saline contained 1 million exosomes. At 24 and 48 h, 10 µL MTT solution (Biotium, California, USA) with a final concentration of 0.5 mg/mL Absorbance was then recorded at a wavelength of 570 nm using a microplate reader (BioTek Synergy H1, BioTek Instruments, Winooski, VT, USA) and the viability rates of the experimental groups were determined by accepting the OD value as 100%.

### Apoptosis and cell cycle analyses

Apoptosis analysis is employed to distinguish living, apoptotic, and necrotic cells. This assay was conducted to assess the impact of varying doses of WJ-MSC-derived small EVs on the apoptosis of PDAC cells. Panc-1 cells were inoculated into a 24-well plate at a density of 50.000 cells per well. Six wells per experimental group were analyzed to ensure experimental replicates. Small EVs were introduced to Panc-1 cells at doses of 4.000, 8.000, and 12.000 EVs per cell. Apoptosis and cell cycyle experiments were conducted using flow cytometry after 24 h. Fluorescein isothiocyanate (FITC)-labeled Annexin V was used in accordance with the protocol of the V Apoptosis Detection Kit, which includes 7-Aminoactinomycin D (7-AAD) [Tonbo Bioscience, Cat. No. 35-6410 (San Diego, United States)]. The cells were subsequently centrifuged at 1000×g for 5 min. A 1× Annexin V binding buffer, included in the kit, was added to each cell pellet at a volume of 500 μL per sample. Next, 100 μL of binding buffer, 5 μL of FITC Annexin V dye, and 5 μL of 7-AAD were added to each pellet. Following a 15-min incubation in the dark at 25 °C, measurements were obtained using a NovoCyte Flow Cytometer, and graphs were generated with NovoExpress Software (1.6.1).

The cell cycle test is used to determine the proportion of stages within the cell cycle based on DNA content. This study assessed the impact of varying doses of WJ-MSC-derived small EVs on the cell cycle of PDAC cells. The Cell Cycle Analysis Kit (THOR-CCK-100, Thorvacs Biotechnology, Turkey) was used in accordance with the manufacturer’s instructions. After preparing the cells as per the protocol, propidium iodide was added and incubated at 37 °C for 30 min. Histograms were generated using NovoExpress Software (1.6.1) after analysis with the NovoCyte Flow Cytometer.

### Gene expression analyses

This analysis was conducted to determine the effect of varying doses of WJ-MSC-derived small EVs on the EMT mechanism and immune gene expression in PDAC cells. Panc-1 cells were seeded in a 24-well plate at a density of 50.000 cells per well. Small EVs were added to the Panc-1 cells at doses of 4.000, 8.000, and 12.000 EVs per cell. Cells were collected from 12 wells per experimental group after 24 h. Total RNA was extracted using Trizol (SERVA, Germany) following the manufacturer’s protocol. Gene expression analysis was performed using 100 ng of RNA for reverse transcription PCR (RT-PCR) on the Lightcycler® 96 system (Roche Diagnostic Systems, Indianapolis, IN). Quantitative RT-PCR (qRT-PCR) was conducted using One-Step GoldNStart TaqGreen RT-qPCR Master Mix. The genes analyzed included Glyceraldehyde 3-phosphate dehydrogenase (*GAPDH*), *CD44* (CD44 Molecule), *CDH1* (E-cadherin), *CLND1* (Claudin 1), *VIM* (Vimentin), *ZEB1* (Zinc finger E-box-binding homeobox 1), Interleukin 1-alpha (*IL-1α*), Interleukin 1-beta (*IL-1β*), Tumor Necrosis Factor Alpha (*TNF-α*), Interleukin 6 (*IL-6*), Interleukin 10 (*IL-10*), and Interferon-γ (*IFN-γ*), all used at a final concentration of 0.1 μM. The gene-specific primer sequences used are provided in Table 1.

**Table 1:** Primers used for qRT-PCR expression analysis

Gene name	Forward sequence	Reverse sequence
*GAPDH*	5′‐GTCTCCTCTGACTTCAACAGCG‐3′	5′‐ACCACCCTGTTGCTGTAGCCAA‐3′
*CD44*	5′- CACACGAAGGAAAGCAGGAC -3′	5′- CCAGAGGTTGTGTTTGCTCC -3′
*CDH1*	5′-TTAGAGGTCAGCGTGTGTGA-3′	5′- CTTCTCCGCCTCCTTCTTCA -3′
*VIM*	5′-CTGCCAACCGGAACAATGAC-3′	5′- TAGTTAGCAGCTTCAACGGC-3′
*ZEB1*	5′-AGGAGCCACAAAAGGACAGT- 3′	5′- TGGGGAATCAGAATCGTTTGC-3′
*CLDN1*	5′- TGCTTGGAAGACGATGAGGT- 3′	5′- GAGCCTGACCAAATTCGTACC-3′
*IL-6*	5′-CTCCACAAGCGCCTTCGGT-3′	5′-GAATCTTCTCCTGGGGGTACTGG-3′
*IL-10*	5′-CCTGCCTAACATGCTTCGAG- 3′	5′-GAGTTCACATGCGCCTTGAT- 3′
*TNF-α*	5′-GCCCATGTTGTAGCAAACCCTC-3′	5′-GGTTATCTCTCAGCTCCACGCC-3′
*IFN-γ*	5′-GCTGTTACTGCCAGGACCC-3′	5′-TTTTCTGTCACTCTCCTCTTTCC-3′
*IL-1α*	5′-TGATCAGTACCTCACGGCTG-3′	5′-TGGTCTTCATCTTGGGCAGT-3′
*IL-1β*	5′-CGAATCTCCGACCACCACTA-3′	5′-AGCCTCGTTATCCCATGTGT-3′

### Immunohistochemistry staining

This analysis was conducted to visualize the effect of CD44, Vimentin and E-cadherin protein expression Panc-1 cells. For Immunohistochemistry (IHC) staining, all cells were cultured on 13 mm uncoated glass coverslips 24 h. After removing the medium, cells were incubated in 4% Paraformaldehyde (SERVA, Germany), 0.1% Triton X-100, and 1% Bovine serum albumin solutions. Then, they were incubated with CD44 antibodies (DF6392, Affinity Biosciences), E-cadherin (E-AB-31261, Elabscience), and Vimentin (E-AB-67478, Elabscience) at a 1:200 dilution in one group one hour. Next, 2-step plus Poly-HRP Anti Rabbit IgG Detection System with DAB Solution (Cat no: E-IR-R215) was used according to the manufacturer protocol. Then, they were incubated with Hematoxylin (SERVA, Germany) dye. The images of the stained samples were captured using a Nicon Eclipse E200 upright microscope.

### Statistical analysis

The data are presented as mean ± SD for various measurements. GraphPad Prism 8.1 (GraphPad Software, San Diego, CA, USA) was utilized to create the graphs and figures. Protein–cytokine quantifications and gene expression analyses were conducted in triplicate (*n* = 3), while viability, cell cycle, and apoptosis assays were performed in six biological replicates (*n* = 6). Statistical analysis was carried out using one-way ANOVA (GraphPad Software, San Diego, CA, USA) followed by Bartlett’s post hoc testing to compare differences among groups. A *p* value of less than 0.05 is considered statistically significant.

## Results

### Characterization of mesenchymal stem cells and small EVs

**Fig. 1:**
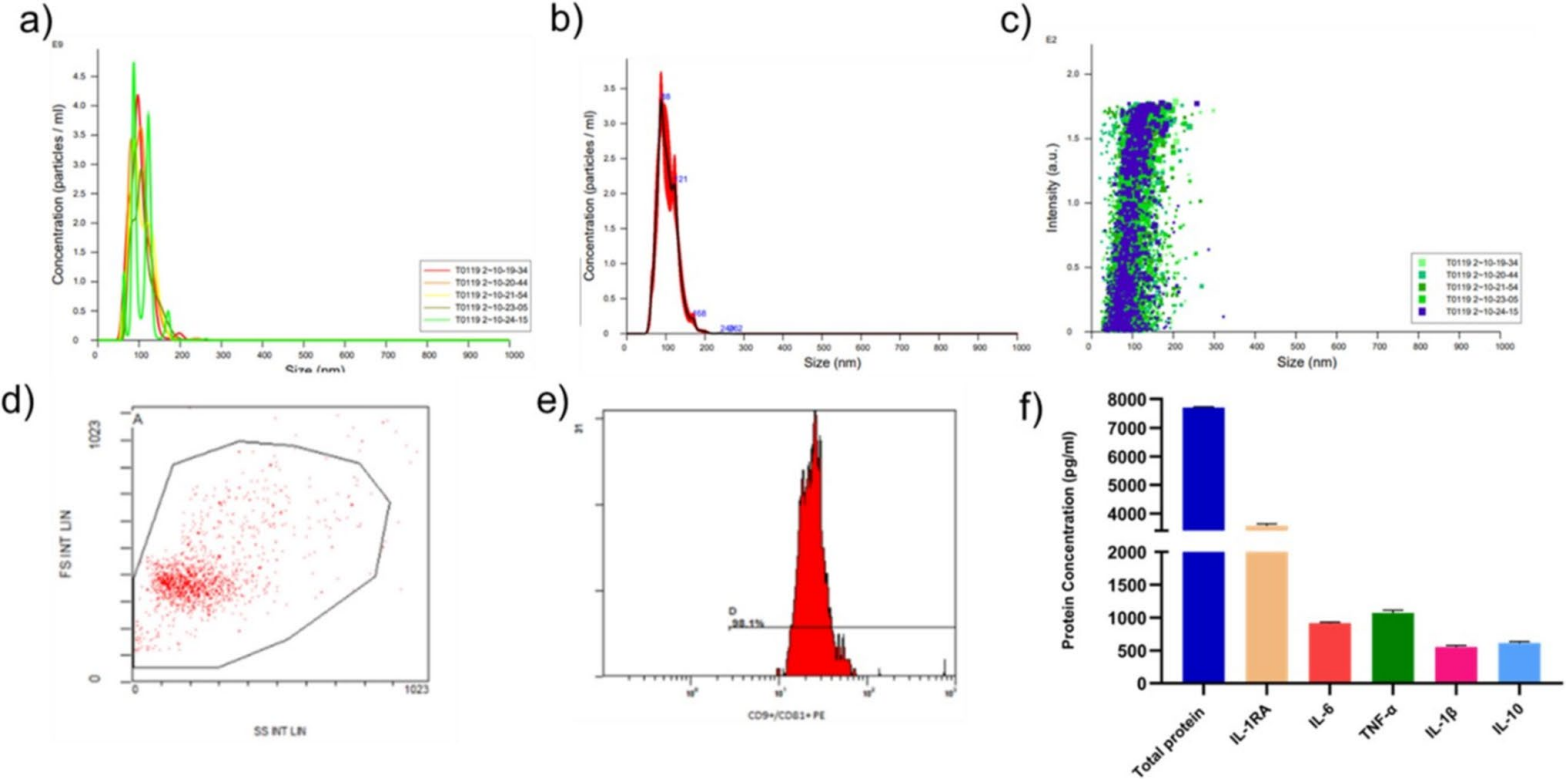
NTA analysis, flow cytometry analysis and protein concentration of small EVs. Graph showing size and concentration per ml in five separate analyses of EVs sample performed by the instrument (**a**). Graph showing the average concentration (**b**). Dot plot of size and concentration of extracellular particles (**c**). Graph showing the passage of the target cell cluster (**d**). Graphical representation of positive cells within the cell cluster (e). Graph shows total protein and cytokine levels (**f**). The results are presented as the mean from three independent experiments (mean ± SD, *n* = 3)

We morphologically characterized the cultured cells as MSCs and observed their characteristic fibroblast-like morphology (Supplementary Fig. 1a). Staining-based distribution and histogram plots illustrated the flow cytometry analysis of MSCs. The analysis included 45.686 cells out of 50.000 stained cells, with an 86.3% passage interval. 99.74% of the stained cells in the passage range expressed CD73, 99.61% expressed CD90, 99.28% expressed CD44, and 99.80% expressed CD105, confirming mesenchymal stem cells characteristic (Supplementary Fig. 1b). We used the NTA device and flow cytometry equipment in this section to monitor the characterization of small EVs. This analysis (Fig. 1a) also determined the size of EVs in nm from five separate measurements of the exosome sample using the NTA device, as well as the number of particles per ml. The NTA results revealed an average EV size of 104.6 ± 1.6 nm, and the graph (Fig. 1b) presented the average concentration. We present a dot plot of the density and size of EV particles (Fig. 1c). A graph that illustrates the gate setting of the target cell cluster was presented in Fig. 1d. CD63 and CD9 surface markers were positive over 98.05% of the time (e). The total protein content of exosomes obtained from 1.5 mL of culture medium was 7713.565 pg/mL. The concentration of the cytokines were found as 3569.204 pg/ml for IL-1RA, 915.833 pg/ml for IL-6, 1071.62 pg/ml for TNF-α, 551.98 pg/ml for IL-1β, and 615.687 pg/ml for IL-10 (Fig. 1f). All experiments for characterization confirmed that the vesicles obtained were small EVs.

### MSC-derived small EVs influence the cell viability and cell cycle of Panc-1 cells

The analyses in this section were performed at 24 and 48 h by MTT method to determine the viability of WJ-MSC-derived exosomes on cancer cells. At the end of the 24th hour, the viability of the Panc-1 cell line was found to be 94% in the group treated with 4000 EVs per cell after the untreated group was accepted as 100%. In the group with 10,000 EVs per cell, 74% was found. Values in this range were observed in the intermediate groups (Supp. Fig. 2a). Similarly, at the end of the 48th hour, the viability of the Panc-1 cell line was found to be 94% in the group treated with 4000 EVs per cell after the untreated group was accepted as 100%. In the group treated with 10,000 EVs per cell, 73% was found. Values in this range were observed in the intermediate groups (Supp. Fig. 2b). It can be stated that WJ-MSC-derived exosomes may reduce viability depending on the amount. It was decided that the initial number of EVs should be 4000 for per cell further experiments.

**Fig. 2:**
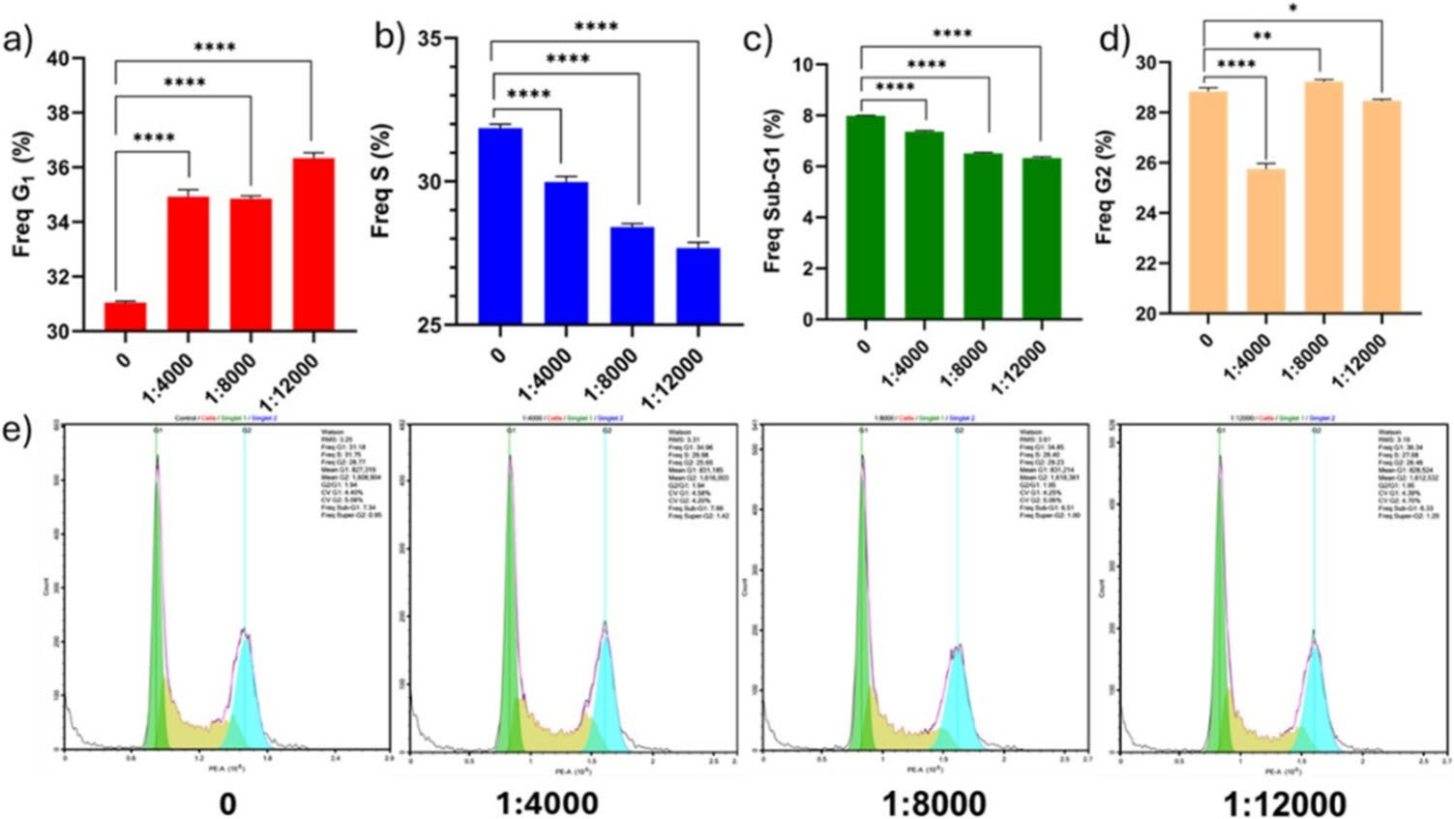
This image shows the effects of MSC-derived small EVs on the cell cycle of Panc-1 cells after the application of 4000, 8000, and 12,000 EVs per cell. Application of MSC-derived EVs at 4000, 8000 and 12,000 EVs per cell for 24 h is shown in G1 (**a**), S (**b**), G2 (**c**), and Sub-G1 (**d**) graphs and histograms (**e**). The results are presented as the mean of six independent experiments (mean ± SD, *n* = 6). Statistical significance is represented by stars: * for *p* < 0.05, ** for *p* < 0.01, *** for *p* < 0.001, and **** for *p* < 0.0001, indicating a significant difference from the control group

In this in-depth flow cytometry study, we looked at how MSC-derived EVs change the cell cycle distribution of Panc-1 cells. Application of MSC-derived EVs at 4000 EVs per cell for 24 h increased the G1 cell population by 3.9% and decreased the S cell population by 1.8% (Fig. 2a, b, e). Application of MSC-derived EVs at 8000 EVs per cell for 24 h increased G1 cell population by 3.8% and decreased S cell population by 3.4% (Fig. 2a, b, e). Application of MSC-derived EVs at 12,000 EVs per cell for 24 h increased G1 cell population by 5.3% and decreased S cell population by 4.2% (Fig. 2a, b, e). These findings suggest a potential induction of cell cycle arrest. Treatment of MSC-derived EVs with 4000, 8000, and 12,000 particles per Panc-1 cell for 24 h resulted in a relative decrease in the Sub-G1 cell population compared to the untreated group (Fig. 2c, e). When Panc-1 cells were treated with MSC-derived EVs for 24 h, the number of cells in G2 phase was lower in the 4000 group compared to the untreated group (Fig. 2d, e). We observed that MSC-derived EVs modulated Panc-1 cells in an inhibitory direction. This can be considered an advantage for therapy of cancer cells.

### MSC-derived EVs influence the apoptosis of Panc-1 cells

**Fig. 3:**
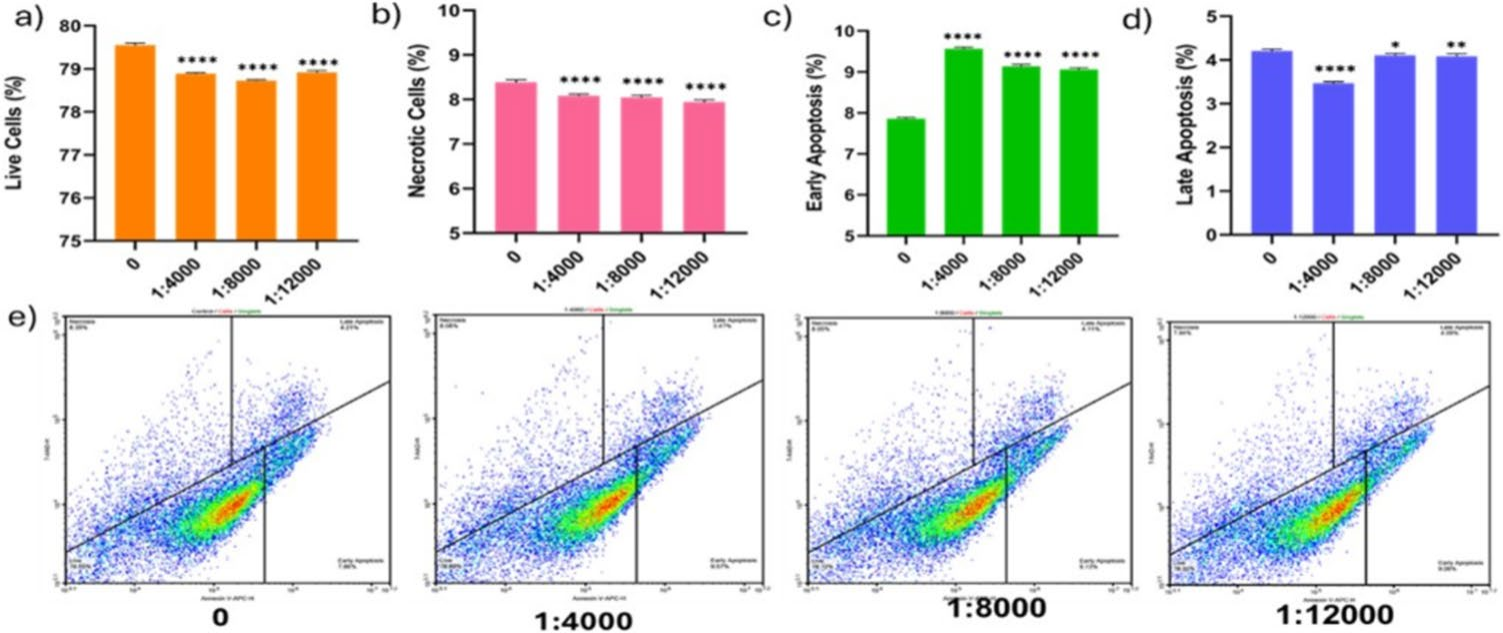
This image shows the effects of MSC-derived small EVs on the viability and apoptosis of Panc-1 cells after the application of 4000, 8000, and 12,000 EVs per cell. The application of MSC-derived EVs at 4000, 8000, and 12,000 EVs per cell for 24 h is shown in the graphs for live cells (**a**), necrotic cells (**b**), early apoptotic cells (**c**), and late apoptotic cells (**d**), as well as in the plots (**e**). The results are presented as the mean from six independent experiments (mean ± SD, *n* = 6). Statistical significance is represented by stars: * for *p* < 0.05, ** for *p* < 0.01, *** for *p* < 0.001, and **** for *p* < 0.0001, indicating a significant difference from the control group

In this study, we looked at how MSC-derived EVs changed the viability, apoptosis, and necrosis of Panc-1 cells. The apoptosis analysis revealed 79.55/8.39 live/necrotic cells in the untreated group, 78.89/8.08 in the 1:4000 groups, 78.72/8.05 in the 1:8000 groups, and 78.92/7.94 in the 1:12,000 groups (Fig. 3a, b, e). EVs exhibited minimal impact on cell viability and necrosis, with changes measuring less than 1%. The apoptosis analysis revealed an early apoptosis/late apoptosis ratio of 7.86%/4.21% in the untreated group, 9.57%/3.47% in the 4000 group, 9.13%/4.11% in the 8000 group, and 9.06%/4.09% in the 12,000 group (Fig. 3c, d, e). At the end of 24 h, we observed that EVs induced early apoptosis by approximately 2%.

### MSC-derived small EVs influence the EMT-related mechanism of Panc-1 cells

**Fig. 4:**
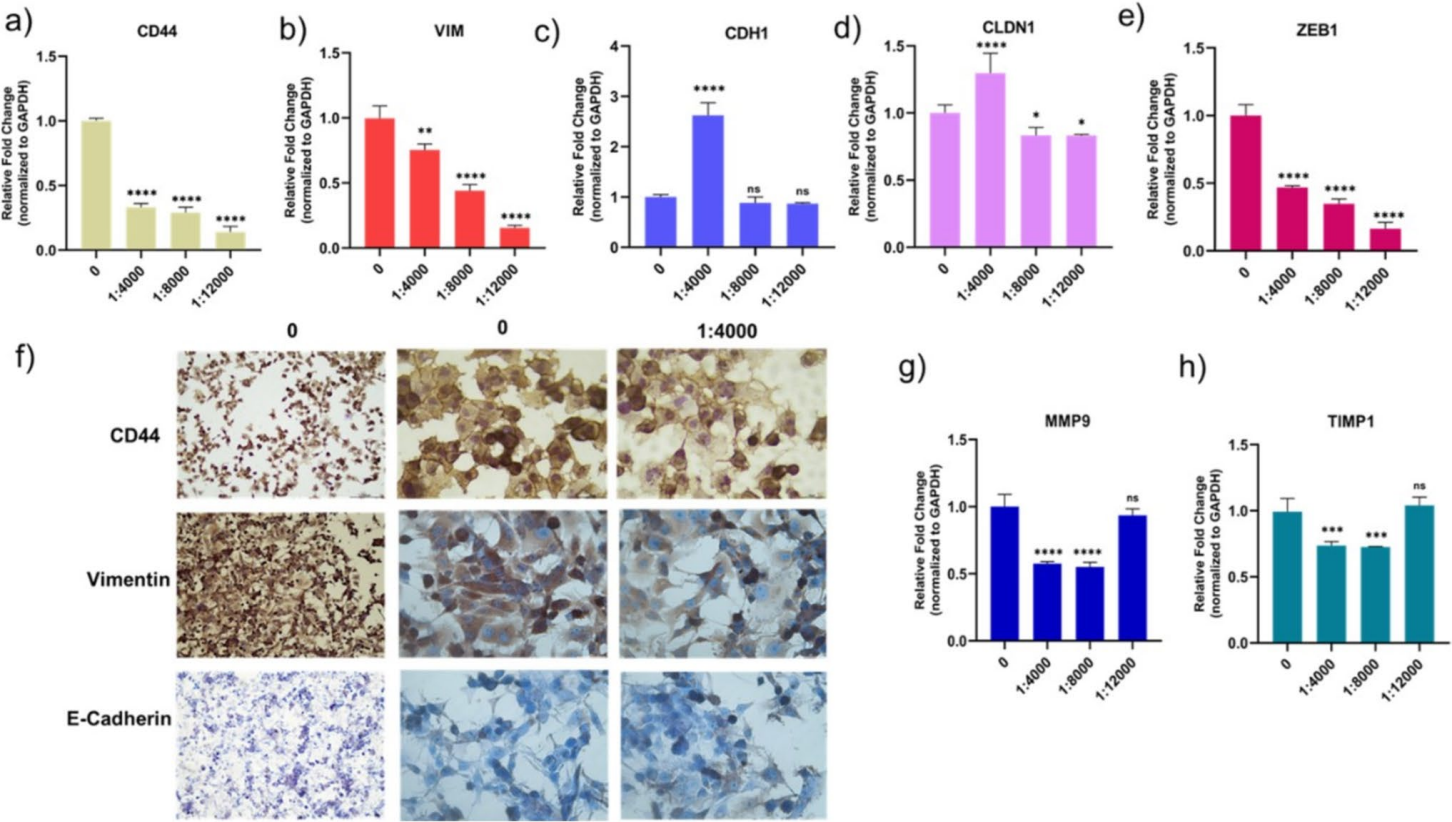
This image shows the changes in gene expression of *CD44, VIM, CDH1, CLDN1, MMP9, TIMP1*, and *ZEB1* in Panc-1 cells treated with MSC-derived small EVs, as well as the IHC staining of CD44, Vimentin, and E-Cadherin proteins. These graphs show the expression changes of *CD44* (**a**), *VIM* (**b**), *ZEB1* (**e**), *CDH1* (**c**), *CLDN1* (**d**), *MMP9* (**g**), and *TIMP1* (**h**) genes in Panc-1 cells by MSC-derived EVs. The expression of CD44, Vimentin, and E-Cadherin proteins in Panc-1 cells are shown in (f). The results are presented as the mean from three independent experiments (mean ± SD, *n* = 3). Statistical significance is represented by stars: * for *p* < 0.05, ** for *p* < 0.01, *** for *p* < 0.001, and **** for *p* < 0.0001, indicating a significant difference from the control group

We examined the expression of the genes CD44, VIM, CDH1, CLDN1, MMP9, TIMP1, and ZEB1 on Panc-1 cells. We linked these genes to the EMT process regulated by EVs derived from MSCs. The expression of CD44, VIM, and ZEB1 genes in Panc-1 cells were downregulated in the presence of MSC-derived Evs (Fig. 4a, b, e). The group with 4000 EVs per cell showed an increase in CDH1 and CLDN1 gene expression (Fig. 4c, d). The group in which cancer cells were treated with 4000 EVs per cell showed a decrease in MMP9 and TIMP1 gene expressions, whereas the group with 12,000 EVs per cell showed no significant change (Fig. 4g, h). These data revealed that MSC-derived EVs effect EMT-related genes and support the epithelial phenotype. Immunohistochemical staining of CD44, vimentin, and E-cadherin proteins was observed in MSC-derived EV-treated group and 4000 treated group (Fig. 4f). The data showed that these proteins were expressed in the Panc-1 control group, which means that Panc-1 cells have a high mesenchymal phenotype.

### MSC-derived small EVs influence the immune regulation-related gene expression of Panc-1 cells

The addition of MSCs-derived EVs to Panc-1 cells resulted in significant alterations in the expression levels of key inflammatory cytokine genes, including *IL-6, IL-10, TNF-α, IFN-γ, IL-1α, and IL-1β* (Table 2). Specifically, in the treated cohorts, a downregulation of *IL-6, TNF-α, IFN-γ, IL-1α,* and *IL-1β* was observed in comparison to the control group, whereas *IL-10* expression was markedly upregulated. These findings indicate that MSC-derived EVs effectively attenuate the pro-inflammatory phenotype of Panc-1 cells while simultaneously enhancing their anti-inflammatory response. This state of immunosuppression is deemed detrimental for tumor cell proliferation and progression.

**Table 2:** The effect of MSC-derived EVs on the fold change and standard deviation of *IL-6, IL-10, TNF-α, IFN-γ, IL-1α,* and *IL-1β* gene expression in Panc-1 cells

Gene name	*IL-6*		*IL-10*		*TNF-α*		*IL-1α*		*IL-1β*		*IFN-γ*	
	Mean	SD	Mean	SD	Mean	SD	Mean	SD	Mean	SD	Mean	SD
0	0.983	0.047	0.990	0.065	1.013	0.070	1.01	0.075	1.006	0.070	1.006	0.080
1:4000	0.133	0.009	5.728	0.528	0.136	0.034	0.152	0.042	0.165	0.015	0.139	0.045
1:8000	0.329	0.026	25.106	2	0.153	0.020	0.150	0.040	0.447	0.035	0.405	0.031
1:12,000	0.577	0.052	36.680	2.516	0.312	0.007	0.194	0.0236	1.043	0.145	0.688	0.040

## Discussion

Recent studies have shown that WJ-MSC-derived EVs may have anticancer effects when engineered and applied to PDAC cells for targeted therapy. Although the naïve application of WJ-MSC-derived small EVs has mostly shown antitumor effects, there is a need to elucidate their underlying mechanisms. In this study, we observed that treatment of Panc-1 cells with 4.000, 8.000, and 12.000 EVs per cell inhibited cell cycle progression, induced early apoptosis, and increased the expression of genes associated with an epithelial phenotype. At the same time, it decreased the expression of pro-inflammatory genes and upregulated IL-10 expression. These findings underscore both the therapeutic promise and associated challenges of employing WJ-MSC-derived EVs, emphasizing the necessity for further optimization studies. A study investigating the effects of the WJ-MSC secretome reported no influence on the proliferation or apoptotic potential of A549 cells [22]. Therefore, the MSC secretome was suggested as a potentially safe option for treating other medical conditions, such as cardiovascular, neurodegenerative, and autoimmune diseases, which may be present in cancer patients. EVs derived from bone marrow MSCs have been shown to promote cancer cell proliferation in vivo, specifically in an MCF-7 breast cancer model [23]. Bone marrow MSC-derived exosomes promoted tumor cell growth by activating the extracellular signal-regulated kinase 1/2 (ERK1/2) pathway in a colorectal cancer model [24]. Exosomes derived from human umbilical cord MSCs have been reported to enhance the proliferation of BxPC3 and Panc-1 pancreatic cancer cells, a phenomenon linked to the upregulation of specific miRNAs [25]. Exosomes containing miR-145-5p, derived from human umbilical cord MSCs, suppressed proliferation, and induced apoptosis in an in vivo pancreatic ductal adenocarcinoma model [18]. The effects of MSC-derived exosomes appear to vary depending on the type of cancer cells. In this study, we found that WJ-MSC-derived EVs did not induce any proliferation and significantly inhibited it in Panc-1 cells.

Yan et al. reported that exosomal miR-16-5p from bone marrow-derived MSCs reduced tumor growth by inducing apoptosis through the regulation of integrin α2 in colorectal cancer [26]. In chronic myeloid leukemia cells, umbilical cord-derived MSC exosomes have been shown to inhibit cell viability and induce apoptosis, with a synergistic effect when combined with Imatinib [27]. WJ-MSC-derived microvesicles were shown to increase early apoptosis and decrease late apoptosis in bladder tumor T24 cells, specifically in the 100 μg microvesicle-treated group [28]. Although the effect of MSC-derived exosomes on apoptosis varies across studies, we demonstrate that MSC-derived small EVs can induce early apoptosis in Panc-1 cells by 2–3% when treated with 4.000, 8.000, and 12.000 EVs.

In regards to the anticancer effects of exosomes derived from MSCs, exosomes secreted by WJ-MSCs have been shown to reduce migration in U87 glioblastoma multiforme cells and carry anti-tumorigenic miRNAs [29]. Yang et al. found that MSC-derived exosomes containing the enzyme matrix metalloproteinase-2 can alter cellular functionalities and reorganize the tumor microenvironment [30]. Bone marrow-derived exosomes have also been shown to promote MG63 and SGC7901 cell growth by activating the Hedgehog signaling pathway [31]. Exosomes derived from umbilical cord MSCs increased the proliferation, invasion, and metastatic potential of gastric cancer cells ex vivo. This was achieved by inducing the EMT process and stimulating Akt protein expression [32]. In parallel with these studies, we propose that exosomes can modulate the EMT phenotype of cancer cells. However, our findings suggest that WJ-MSC-derived small EVs promote the epithelial phenotype while decreasing the mesenchymal phenotype.

Exosomes can also act as antigen carriers to stimulate innate and adaptive immune responses, serving as immunomodulators [33]. IDO-1-containing exosomes secreted by MSCs suppress the immune response by decreasing IFN-γ expression in dendritic cells and NK cells [19]. In addition, exosomes derived from MSCs have been shown to reduce immune responses by inducing IL-10 secretion and increasing the number of regulatory T cells [34]. MSC-derived exosomes have also been reported to promote pancreatic cancer progression and growth by inducing the reversible polarization of macrophages [35]. We propose that WJ-MSC-derived small EVs suppress immune responses by decreasing the expression of pro-inflammatory genes and increasing IL-10 expression.

Our findings confirm that WJ-MSC-derived EVs exert both tumor-suppressive and immunomodulatory effects in PDAC cells. While these dual properties may initially appear contradictory, they reflect the complex bioactivity of EV cargo and highlight the need for context-specific therapeutic strategies. On one hand, the ability of EVs to suppress proliferation, arrest the cell cycle, and modulate EMT markers underscores their anticancer potential. On the other hand, the observed upregulation of anti-inflammatory cytokines (e.g., IL-10) and downregulation of pro-inflammatory mediators suggest an immunosuppressive effect, which may have unintended consequences in immunocompetent hosts. This duality carries significant implications for clinical translation. In certain clinical contexts—particularly those characterized by tumor-associated inflammation—the immunomodulatory effects of MSC-EVs might confer added benefit. However, in patients undergoing immune-based therapies, or those with intact antitumor immunity, these effects could pose risks by dampening immune surveillance. Therefore, unmodified MSC-EVs may be better suited for use as delivery vehicles rather than as standalone therapeutic agents.

Supporting this view, a recent Phase I clinical trial (NCT03608631) is investigating the use of engineered exosomes loaded with KRASG12D siRNA in metastatic PDAC. Such approaches, which preserve the targeting capacity of EVs while minimizing off-target immunological effects, may offer a more clinically viable path forward. Further in vivo studies and functional profiling of EV cargo are essential to optimize the therapeutic index of MSC-derived vesicles.

A limitation of this study is its reliance on an in vitro 2D culture model, which does not fully replicate the complex tumor microenvironment (TME) of PDAC. The dense desmoplastic stroma, immune heterogeneity, and altered extracellular matrix composition in vivo may significantly influence the behavior and therapeutic efficacy of MSC-derived EVs. As such, future investigations using orthotopic animal models or patient-derived xenografts are warranted to assess the translational potential of these findings in a more physiologically relevant setting.

## Conclusion

MSC-derived small EVs or exosomes can be genetically modified to either enhance their antitumor effects or to carry a variety of anticancer agents. These carriers offer advantages such as low immunogenicity, tumor tropism, rapid isolation, and precise targeting. Significant progress has been obtained in MSC exosome-based therapies, particularly in the treatment of pancreatic cancer. However, clinical applications of these therapies face challenges, and a better understanding of the interactions between MSC exosomes and tumor cells is needed. The potential pro-tumorigenic effects of MSC-derived EVs necessitate thorough safety evaluations prior to clinical applications Considering these factors, MSC exosome-based therapies emerge as a promising option for pancreatic cancer treatment.

## Supplementary Information

**Supplementary Figure 1**. Morphological and immunophenotypic characterization of MSCs grown in the laboratory. Fibroblast-like morphology of ADMSCs under light microscopy (a) Immunophenotypic characterization of MSCs was determined by flow cytometer assay (b) CD90, CD73, CD44 and CD105 expressions in all independent experimental groups are shown as histograms.

**Supplementary Figure 2**. The effects of 4000–10000 small EVs treated with per cells on the viability of Panc-1 cells at 24 (a) and 48 (b) hours. Values are expressed as mean ± SD with n = 6. Statistical significance is represented by stars: * for p < 0.05, ** for p < 0.01, *** for p < 0.001, and **** for p < 0.0001, indicating a significant difference from the control group.

Below is the link to the electronic supplementary material.

Supplementary file1 (JPG 124 KB)

Supplementary file2 (JPG 329 KB)

## Data Availability

No datasets were generated or analyzed during the current study.
