# Supplementary figures and images for: Dual facets of MSC-derived small EVs: regulatory insights into antitumor mechanisms in pancreatic ductal adenocarcinoma
Source: Med Oncol. 2025 Apr 10;42(5):158. doi: 10.1007/s12032-025-02713-5 (PMC11985665; doi:10.1007/s12032-025-02713-5)

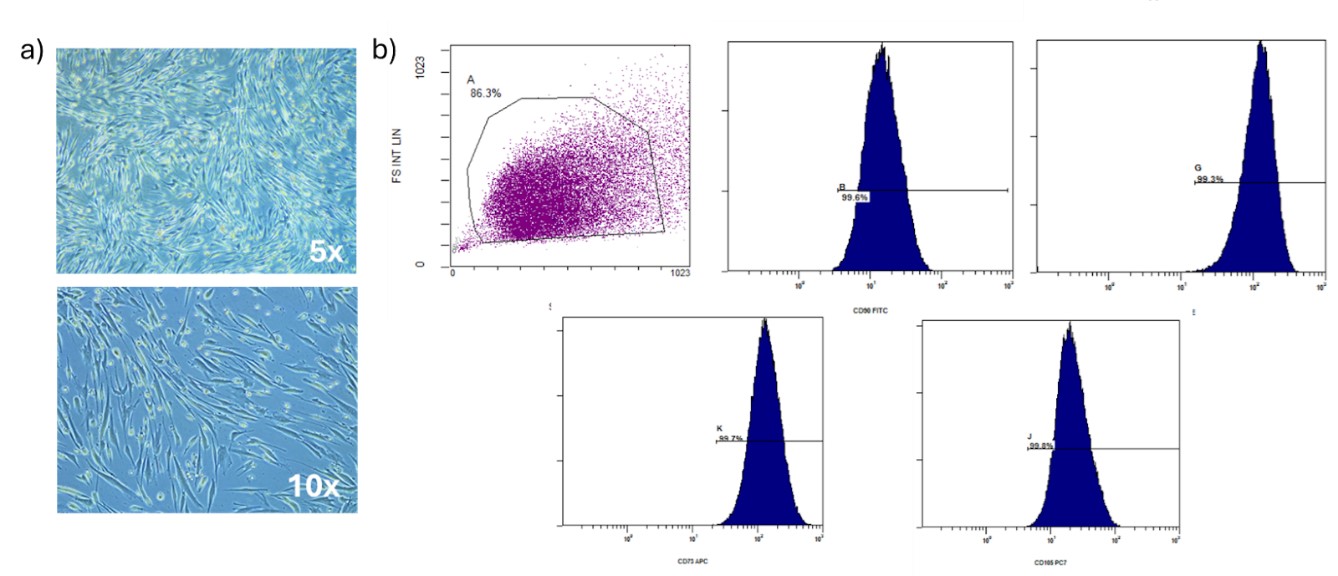

Supplement: Supplementary file 1 — Supplementary file1 (JPG 124 KB) [file 12032_2025_2713_MOESM1_ESM.jpg]

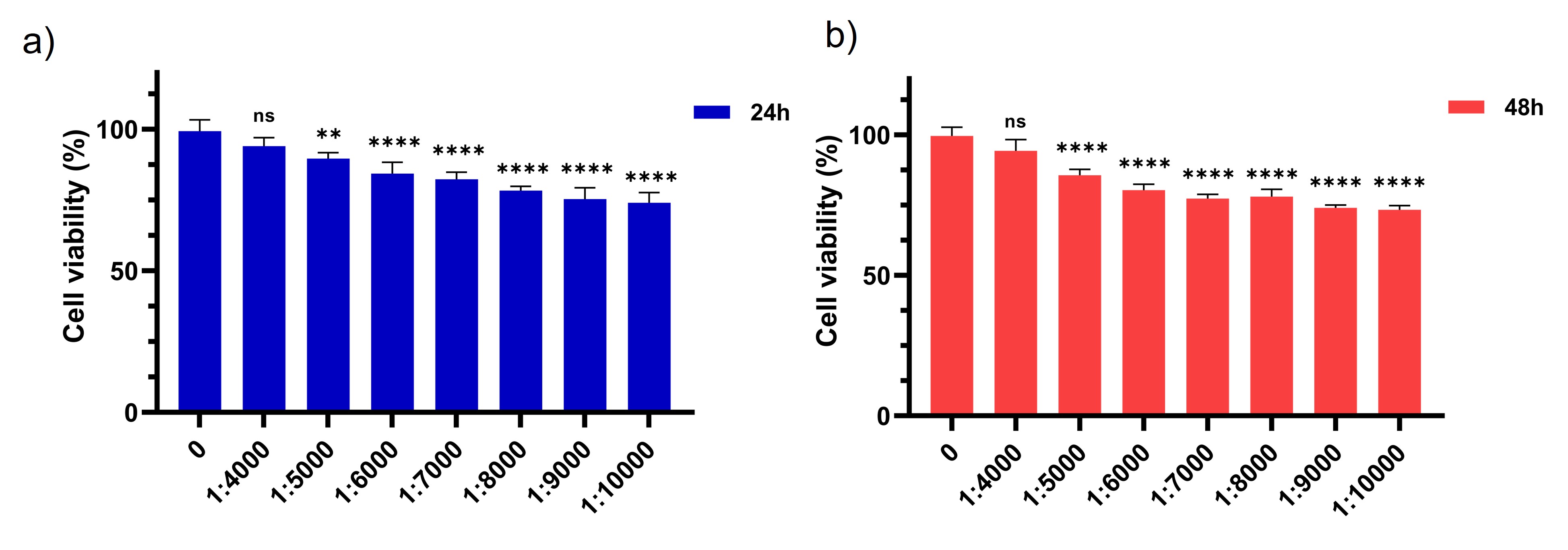

Supplement: Supplementary file 2 — Supplementary file2 (JPG 329 KB) [file 12032_2025_2713_MOESM2_ESM.jpg]
